# Supplementary material for: HIVprotI: an integrated web based platform for prediction and design of HIV proteins inhibitors
Source: J Cheminform. 2018 Mar 9;10:12. doi: 10.1186/s13321-018-0266-y (PMC5845081; doi:10.1186/s13321-018-0266-y)
Supplement: Supplementary file 2 — Additional file 2. Source code of HIVProtI web server. [file 13321_2018_266_MOESM2_ESM.zip › HIVprotI_Source-code/hivprotI/weka-3-6-6/doc/weka/estimators/MahalanobisEstimator.html]

MahalanobisEstimator


---


|  |  |  |
| --- | --- | --- |
| **Overview** \| **Package** \| **Class** \| **Tree** \| **Deprecated** \| **Index** \| **Help** \| **Weka's home** | |  |
| **PREV CLASS**   **NEXT CLASS** | **FRAMES**    **NO FRAMES**     **All Classes** |  |
| SUMMARY: NESTED \| FIELD \| CONSTR \| METHOD | DETAIL: FIELD \| CONSTR \| METHOD |  |


---


## weka.estimators Class MahalanobisEstimator

```
java.lang.Object
  weka.estimators.Estimator
      weka.estimators.MahalanobisEstimator
```

**All Implemented Interfaces:**: java.io.Serializable, java.lang.Cloneable, CapabilitiesHandler, OptionHandler, RevisionHandler, IncrementalEstimator

---

``` public class MahalanobisEstimator extends Estimator implements IncrementalEstimator ```

Simple probability estimator that places a single normal distribution
over the observed values.

**Version:**
:   $Revision: 5540 $

**Author:**
:   Len Trigg (trigg@cs.waikato.ac.nz)

**See Also:**: Serialized Form

---

**Constructor Summary**

`MahalanobisEstimator(Matrix covariance,
double constDelta,
double valueMean)`
  
          Constructor


| **Method Summary** | |
| --- | --- |
| `void` | `addValue(double data, double weight)`             Add a new data value to the current estimator. |
| `Capabilities` | `getCapabilities()`             Returns default capabilities of the classifier. |
| `double` | `getProbability(double data)`             Get a probability estimate for a value |
| `java.lang.String` | `getRevision()`             Returns the revision string. |
| `static void` | `main(java.lang.String[] argv)`             Main method for testing this class. |
| `java.lang.String` | `toString()`             Display a representation of this estimator |

**Methods inherited from class weka.estimators.Estimator**

`addValues, addValues, addValues, addValues, buildEstimator, buildEstimator, clone, debugTipText, equals, forName, getDebug, getOptions, listOptions, makeCopies, makeCopy, setDebug, setOptions, testCapabilities`

**Methods inherited from class java.lang.Object**

`getClass, hashCode, notify, notifyAll, wait, wait, wait`

**Constructor Detail**

### MahalanobisEstimator

```
public MahalanobisEstimator(Matrix covariance,
                            double constDelta,
                            double valueMean)
```

:   Constructor

    **Parameters:**: `covariance` -: `constDelta` -: `valueMean` -


**Method Detail**

### addValue

```
public void addValue(double data,
                     double weight)
```

:   Add a new data value to the current estimator. Does nothing because the
    data is provided in the constructor.

    :   **Specified by:**: `addValue` in interface `IncrementalEstimator` **Overrides:**: `addValue` in class `Estimator`
    :   **Parameters:**: `data` - the new data value: `weight` - the weight assigned to the data value

---


### getProbability

```
public double getProbability(double data)
```

:   Get a probability estimate for a value

    :   **Specified by:**: `getProbability` in class `Estimator`
    :   **Parameters:**: `data` - the value to estimate the probability of **Returns:**: the estimated probability of the supplied value

---


### toString

```
public java.lang.String toString()
```

:   Display a representation of this estimator

    :   **Overrides:**: `toString` in class `java.lang.Object`

---


### getCapabilities

```
public Capabilities getCapabilities()
```

:   Returns default capabilities of the classifier.

    :   **Specified by:**: `getCapabilities` in interface `CapabilitiesHandler` **Overrides:**: `getCapabilities` in class `Estimator`
    :   **Returns:**: the capabilities of this classifier **See Also:**: `Capabilities`

---


### getRevision

```
public java.lang.String getRevision()
```

:   Returns the revision string.

    :   **Specified by:**: `getRevision` in interface `RevisionHandler`
    :   **Returns:**: the revision

---


### main

```
public static void main(java.lang.String[] argv)
```

:   Main method for testing this class.

    :   **Parameters:**: `argv` - should contain a sequence of numeric values


---


|  |  |  |
| --- | --- | --- |
| **Overview** \| **Package** \| **Class** \| **Tree** \| **Deprecated** \| **Index** \| **Help** \| **Weka's home** | |  |
| **PREV CLASS**   **NEXT CLASS** | **FRAMES**    **NO FRAMES**     **All Classes** |  |
| SUMMARY: NESTED \| FIELD \| CONSTR \| METHOD | DETAIL: FIELD \| CONSTR \| METHOD |  |


---
